# Toward Hydrogen‐Free and Dendrite‐Free Aqueous Zinc Batteries: Formation of Zincophilic Protective Layer on Zn Anodes

**DOI:** 10.1002/advs.202104866

**Published:** 2022-01-06

**Authors:** Lin Hong, Liang‐Yu Wang, Yuling Wang, Xiuming Wu, Wei Huang, Yongfeng Zhou, Kai‐Xue Wang, Jie‐Sheng Chen

**Affiliations:** ^1^ School of Chemistry and Chemical Engineering State Key Laboratory of Metal Matrix Composites Shanghai Jiao Tong University 800 Dongchuan Road Shanghai 200240 P. R. China

**Keywords:** dendrite growth, hydrogen evolution reaction, Sb‐modified layers, Zn metal anodes

## Abstract

Rechargeable aqueous Zn‐ion batteries (ZIBs) are regarded as one of the most promising devices for the next‐generation energy storage system. However, the uncontrolled dendrite growth on Zn metal anodes and the side hydrogen evolution reaction, which has not yet been well considered, hinder the practical application of these batteries. Herein, a uniform and robust metallic Sb protective layer is designed based on the theoretic calculation and decorated on Zn plate via in situ replacement reaction. Compared with the bare Zn plate, the as‐prepared Zn@Sb electrode provides abundant zincophilic sites for Zn nucleation, and homogenizes the electric field around the Zn anode surface, both of which promote the uniform Zn deposition to achieve a dendrite‐free morphology. Moreover, the Gibbs free energy (∆*G*_H_) calculation and in situ characterization demonstrate that hydrogen evolution reaction can be effectively suppressed by the Sb layer. Consequently, Sb‐modified Zn anodes exhibit an ultralow voltage hysteresis of 34 mV and achieve excellent cycling stability over 1000 h with hydrogen‐ and dendrite‐free behaviors. This work provides a facile and effective strategy to suppress both hydrogen evolution reaction and dendrite growth.

## Graphical abstract

A uniform and robust metallic Sb protective layer is decorated on Zn plate via in situ replacement reaction. With rich zincophilic sites for Zn nucleation, improved electrolyte wettability and homogenized electric field, the Sb layer promotes the uniform Zn deposition with a dendrite‐free morphology. Moreover, the Gibbs free energy calculation and in situ characterization demonstrate that hydrogen evolution reaction can be effectively suppressed by the Sb layer.

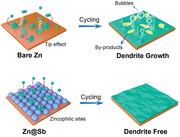

## 1 Introduction

Metallic Zn anodes, as highly promising candidates for aqueous batteries, have recently attracted extensive interest due to their unique features, including intrinsic safety, low cost, high theoretical capacity (820 mAh g^–1^), and moderate redox potential (−0.762 V vs standard hydrogen electrode).^[^ ^1^ ^]^ However, Zn metal anodes in mild electrolyte still suffer from hydrogen evolution reaction and Zn dendrite growth, directly compromising the reversibility and cycling life of the batteries. According to the Pourbaix diagram,^[^ ^2^ ^]^ the reduction of Zn^2+^ in mildly acidic electrolyte is inevitably accompanied with H_2_ evolution through H_2_O decomposition, due to the narrow electrochemical stability window (1.23 V) of aqueous solution and low standard electrode potential of Zn^2+^/Zn. The continuous hydrogen evolution reaction would locally produces OH^–^ ions, thus inducing the consumption of the electrolyte and the formation of by‐products, such as Zn_4_(OH)_6_SO_4_·*x*H_2_O.^[^ ^3^ ^]^ The formation of these by‐products increases the contact surface area between the Zn anode and electrolyte and in turn exacerbates the hydrogen evolution reaction. The gas evolution also leads to a sharp increase of the internal pressure in the sealed batteries, and thereby results in safety hazards. However, the hydrogen evolution reaction has long been overlooked and its characterization cannot well reflect the real operation of batteries so far. Further in situ and quantitative characterizations are highly desired to reveal the correlation between hydrogen evolution reaction and electrochemical performance.

Up to now, various strategies, such as the rationalization of electrolyte formulations,^[^ ^4^ ^]^ the addition of electrolyte additives,^[^ ^5^ ^]^ and the adoption of highly concentrated electrolyte,^[^ ^6^ ^]^ have been developed to alleviate the corrosion issue. Progress has been made in the stabilization of Zn metal interface. However, the uncontrolled dendrite growth during long‐term cycling is still a critical challenge. In turn, the disordered, loose, and porous Zn dendrites generated on the anode interface would accelerate the hydrogen evolution by providing more reaction sites. Therefore, to develop high‐performance rechargeable ZIBs, the issues of both hydrogen evolution and uncontrolled dendrite growth on Zn anode should be simultaneously addressed. The growth of Zn dendrite originated from inhomogeneous Zn nucleation and crystal growth is strongly associated with the surface property of Zn anode. To this end, the introduction of zincophilic sites or interfaces is an effective way to enhance the homogeneity of Zn deposition on Zn anode.^[^ ^7^ ^]^ The zincophilic sites can reinforce the interfacial interaction between Zn^2+^ ions and electrode, consequently reducing the nucleation energy barrier and homogenizing the ion distribution and electric field. For example, zincophilic nitrogen‐doped vertical graphene could induce the uniform Zn nucleation and thereby inhibit the dendrite growth.^[^ ^7b^ ^]^ Sn‐modified 3D carbon felt anodic host provided abundant active sites for Zn nucleation to induce uniform Zn plating/stripping behavior, lowering the nucleation overpotential of Zn and reducing the hydrogen evolution.^[^ ^8^ ^]^ Therefore, the development of zincophilic Zn anodes with dendrite‐free and hydrogen‐free behavior is of great significance to develop rechargeable aqueous ZIBs with long‐term cycling stability.

As indicated by density functional theory (DFT) calculation, the Sb layer has a lower binding energy of −0.71 eV toward Zn atom than that of bare Zn plate (−0.63 eV), indicating the good Zn affinity of Sb. Moreover, the Sb interface has a high hydrogen adsorption Gibbs free energy (∆*G*_H_) of 1.37 eV, which could suppress the hydrogen evolution reaction. Herein, guided by the theoretic calculation, a homogeneous and robust metallic Sb protective layer was prepared on commercial Zn plate through in situ replacement reaction between Zn and Sb^3+^. As demonstrated by morphology evolution and in situ gas detection, the Sb layer on Zn metal acts as both nucleation sites and hydrogen evolution inhibitor, achieving a dendrite‐free and hydrogen‐free Zn metal anode. Consequently, the as‐fabricated Zn@Sb anodes deliver high plating/stripping reversibility and ultralong cycling lifespan at a high current density of 5.0 mA cm^−2^.

## 2 Results and Discussion

**Figure 1:**
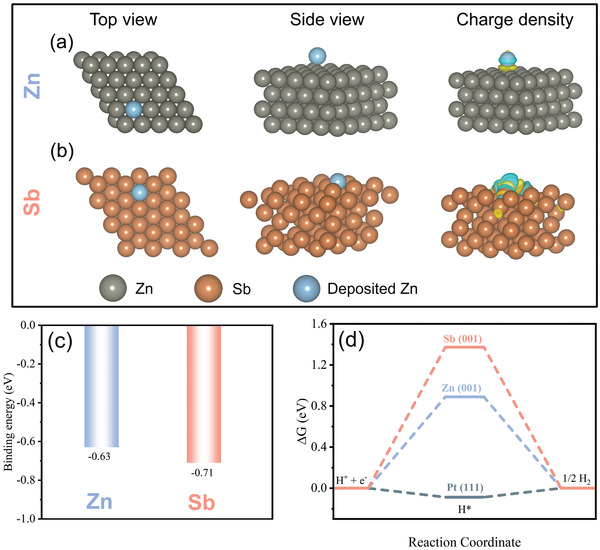
a,b) Adsorption configuration, charge density difference map, and c) corresponding binding energy of Zn adsorbed on Zn (001) and Sb (001) facets. d) Gibbs free energy diagram for hydrogen evolution reaction on Zn (001) and Sb (001).

The modification of Zn anode by the Sb layer was based on the theoretic calculations. DFT calculations were performed to illustrate the adsorption behaviors of a Zn atom on Zn (001) (**Figure** **1**a) and Sb (001) facets (Figure 1b). Binding energy (*E*_b_) is used as the criterion to evaluate the Zn affinity of these surfaces. The *E*_b_ of Sb (001) surface toward Zn atom is −0.71 eV, while that of Zn (001) surface is −0.63 eV (Figure 1c). A lower *E*_b_ suggests that the interaction between Zn atom and Sb (001) is stronger, and Zn prefers to deposit on Sb (001) facet compared to Zn (001) facet. As revealed by charge density difference plots, the electron of deposited Zn atom on the Sb (001) surface is more delocalized, contributing to the enhancement of the metallic bond between deposited Zn and the electrode. The potential of Sb on the inhibition of hydrogen evolution reaction was evaluated by the hydrogen adsorption ∆*G*_H_. Theoretically, large ∆*G*_H_ which indicates the weak binding of the metal surface in the adsorption of hydrogen would suppress the hydrogen release process. As illustrated in Figure 1d, ∆*G*_H_ for Pt (111) and Zn (001) are −0.09 and 0.89 eV, respectively, consistent with the previous reports.^[^ ^3^, ^9^ ^]^ While for Sb (001), a much larger ∆*G*_H_ of 1.37 eV is obtained, indicating that Sb could suppress the hydrogen evolution reaction. Furthermore, the charge redistribution on Zn (001) and Sb (001) with H adsorbed was calculated (Figure S1, Supporting Information). The binding between H atom and Sb atom along out‐plane direction is relatively weak than that between H atom and Zn atoms, further demonstrating the low possibility of hydrogen evolution endowed by Sb layer. Based on the theoretic calculation, the formation of a thin Sb layer with abundant zincophilic and weak hydrogen adsorption sites is proposed as effective strategy to inhibit the Zn dendrite growth and hydrogen evolution.

**Figure 2:**
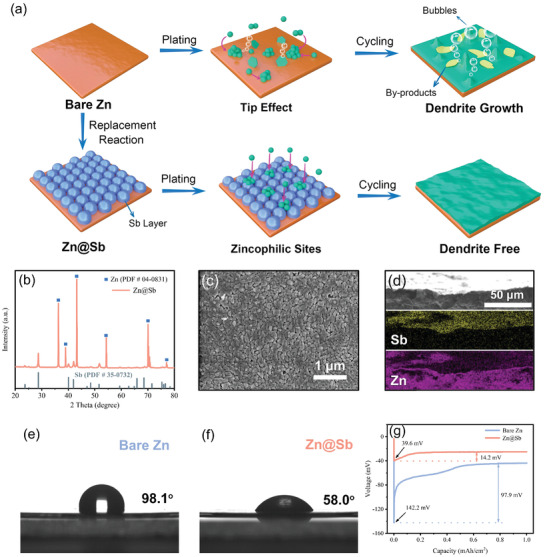
a) Schematic illustration of Zn deposition process on bare Zn and Zn@Sb. b) XRD pattern, c) SEM image, d) cross‐sectional SEM image and corresponding elemental mapping of Zn@Sb. Contact angles of electrolyte on e) bare Zn and f) Zn@Sb. g) Nucleation overpotentials of bare Zn and Zn@Sb at 1.0 mA cm^–2^.

Motivated by the above theoretic analyses, Sb was coated onto Zn foil anodes through a facile chemical displacement reaction (3 Zn + 2 SbCl_3_ → 2 Sb + 3 ZnCl_2_) by immersing Zn plates into SbCl_3_ ethanol solution for 5 min (**Figure** **2**a). In principle, a species possessing higher redox potential can be replaced by that with lower redox potential in solution.^[^ ^10^ ^]^ The redox potential of Zn^2+^/Zn is −0.762 V (vs standard hydrogen electrode) is much lower than that of Sb^3+^/Sb (‐0.51 V vs. standard hydrogen electrode), ensuring the replacement of Sb^3+^ by Zn. The modified Zn foils were washed with ethanol several times and then dried naturally to obtain the Zn@Sb anodes. X‐ray diffraction (XRD) pattern of Zn@Sb anodes shows the characteristic peaks of Zn (JCPDS No. 04–0831) (Figure 2b). The peaks at 23.7^o^, 28.7^o^, 40.0^o^, 41.9^o^, and 51.6° can be indexed to the (003), (012), (104), (110), and (202) planes of Sb (JCPDS No. 35–0732), respectively, demonstrating the successful formation of Sb on Zn foils. Scanning electron microscopy (SEM) observation shows that a coating layer composed of stacked Sb nanoparticles with particle size of ≈100 nm is generated on the surface of Zn foil (Figure 2c). Meanwhile, elemental mapping on the cross‐section of the electrode demonstrates the formation of Sb layer on the surface of Zn@Sb anode (Figure 2d). All the above results confirm the successful modification of the Zn surface by Sb.

The wettability of anodes was characterized by contact angle tests with electrolyte. After the formation of the Sb layer, the contact angle of Zn@Sb is significantly decreased from 98.1^o^ (bare Zn) to 58.0^o^ (Figure 2e,f), indicating the wettability of anodes is enhanced by the Sb coating. The enhanced wettability would promote the homogeneous dispersion of electrolyte over the anode surface. Correspondingly, the calculated surface free energy of Zn@Sb is 63.9 mN m^−1^, larger than that of bare Zn (34.7 mN m^−1^) (Figure S2, Supporting Information). The high surface free energy of Zn@Sb may induce the uniform distribution of Zn^2+^ ions and consequently achieve smooth Zn deposition. Overpotential is an essential parameter to assess the Zn deposition kinetics. The nucleation overpotential and plateau overpotential of Zn@Sb are only 39.6 and 25.4 mV at 1.0 mA cm^–2^, respectively, while the corresponding values of bare Zn are 142.2 and 44.3 mV, respectively (Figure 2g). The reduced polarization of Zn@Sb anodes indicates the low nucleation energy barrier, which is strongly associated with the good affinity of Zn@Sb anodes. The zincophilic sites introduced by the Sb layer on Zn@Sb could enhance the interfacial interaction between Zn^2+^ ions and electrode, thus reducing the required energy for nucleation and growth.

**Figure 3:**
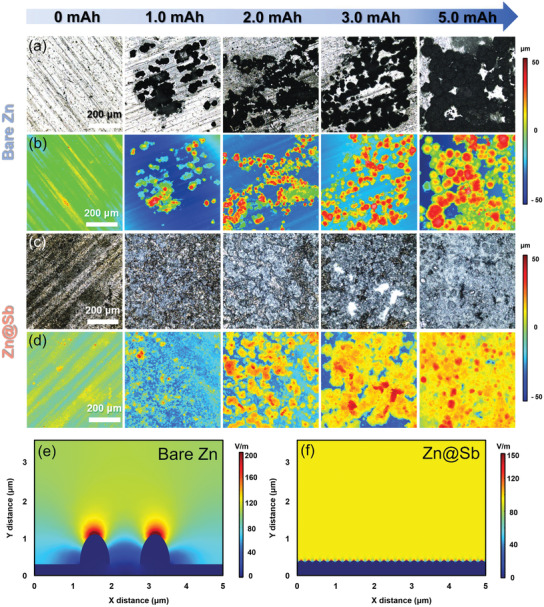
a,c) CLSM optical images and b,d) corresponding height images of Zn deposition at a current density of 0.5 mA cm^–2^ with different capacities on bare Zn and Zn@Sb. Simulated electric field distributions on e) bare Zn and f) Zn@Sb.

The morphology evolution of Zn deposition on bare Zn and Zn@Sb with loading capacities ranging from 1.0 to 5.0 mAh cm^–2^ was investigated by confocal laser scanning microscope (CLSM). For bare Zn, distinct large particle‐shaped Zn dendrites are observed on the Zn anode even at the initial stage of Zn plating (1.0 mAh cm^–2^) (**Figure** **3**a,b). The formation of these large particles is attributed to the limited amount of nucleation sites and the poor wettability of the Zn surface, which can further induce the continuous accumulation of Zn^2+^ ions and charges and consequent uneven Zn deposition. Increasing the deposition capacity, further growth of the particles is observed. When the capacity increases to 5.0 mAh cm^–2^, large Zn protrusions with altitude intercept over 90 µm cover almost the whole Zn surface, attributing to the uncontrolled growth of Zn dendrites. The continuous growth of the dendrites would puncture the separator and consequently lead to the failure of the battery. After the formation of Sb layer, homogenous Zn deposits are observed on the surface of Zn@Sb after the initial plating process at 1.0 mAh cm^–2^ (Figure 3c,d). The homogeneous deposition is attributed to the increased wettability of the Zn surface and the presence of large number of zincophilic nucleation sites, which could regulate the uniform Zn^2+^ ion distribution and consequently induce the formation of film‐like Zn deposition instead of large Zn dendrites. Further increasing the plating capacity to 5.0 mAh cm^–2^, a relatively flat and dense Zn deposition layer with altitude intercept lower than 43 µm is formed on the surface of the Zn@Sb anode.

Besides the Zn affinity, the electric field distribution over the anode surface is another significant influence factor on the nucleation and deposition of Zn. To investigate the effect of Sb layer on interfacial electric field, finite element simulation was performed using COMSOL Multiphysics. Owing to the poor Zn affinity, Zn^2+^ ions tend to deposit randomly on the surface of bare Zn electrode and form irregular protuberances (Figure 3e). These protuberances strengthen the surrounding electric field intensity, driving the nucleation of the excessive Zn^2+^ ions around the tips and consequently promoting the formation of Zn dendrites. The formation of surface Sb layer can dramatically decrease the field intensity of electrode surface from 256.3 to 130.6 V m^–1^ and uniformize the surface electric field in the whole area (Figure 3f). Under the uniform electric field, the distribution of Zn^2+^ ions and charges on electrode surface can be homogenized, contributing to the uniform Zn plating and dendrite‐free morphology.

**Figure 4:**
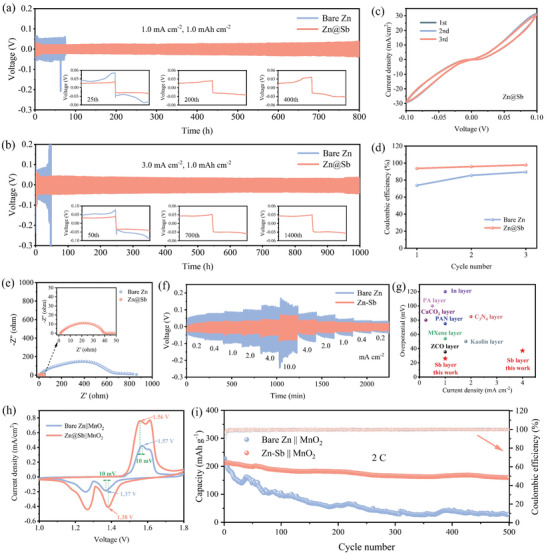
Cycling performance of the Zn symmetric cells at a) 1.0 mA cm^–2^ and b) 3.0 mA cm^–2^ for 1.0 mAh cm^–2^. c) CV curves of the symmetric Zn@Sb cells measured at 0.1 mV s^−1^ and d) corresponding Coulombic efficiency. e) Nyquist plots of the symmetric Zn and Zn@Sb cells. f) Rate performance of symmetric cells at current densities from 0.2 to 10 mA cm^–2^. g) Comparison of voltage hysteresis of Zn@Sb with those Zn anodes reported in the literature. Electrochemical performance of Zn||MnO_2_ full cells: h) CV curves at 0.08 mV s^–1^ and i) long‐term cycling stability at 2 C.

The cycling performance of Zn and Zn@Sb anodes was evaluated by symmetric cells. At 1.0 mA cm^–2^ for 0.5 mAh cm^–2^ (Figure S3, Supporting Information), the Zn@Sb anode exhibits much longer lifetime and lower voltage hysteresis than bare Zn, attributing to the even Zn deposition induced by zincophilic sites. Cycled at 1.0 mA cm^–2^ for 1.0 mAh cm^–2^ (**Figure** **4**a), significant voltage oscillation is observed for bare Zn and consequent short‐circuit failure occurs after only 74 h. The premature failure is possibly associated with the uncontrolled dendrite growth and the consequent puncture of the separator. On the contrary, the cell with the Zn@Sb anode shows prolonged lifespan of over 800 h with low polarization voltage of ≈26 mV under the identical condition. When the current density is increased to 3.0 mA cm^–2^ (Figure 4b), the cell with the Zn@Sb anode can still maintain stable stripping/plating cycles for 1000 h with a stable voltage hysteresis of ≈34 mV. Even at a high current density of 5.0 mA cm^–2^, a ultralong lifespan of over 900 h is still achieved for the Zn@Sb anode (Figure S4, Supporting Information), superior to most of the recently reported Zn anodes (Table S1, Supporting Information).

To investigate the reversibility of the bare Zn and Zn@Sb anodes in the plating/stripping process, cyclic voltammetry (CV) tests of symmetric cells were carried out. As exhibited in Figure 4c and Figure S5 (Supporting Information), Zn@Sb shows larger peak area and higher current intensity than bare Zn, demonstrating the enhanced plating/stripping kinetics endowed by the Sb layer. Moreover, the CV curves of Zn@Sb are well overlapped from the first cycle to the third cycle, indicating the high reversibility of the plating/stripping process. Correspondingly, the Coulombic efficiency of Zn@Sb reaches over 97.7%, much higher than that of bare Zn (Figure 4d). The inferior reversibility of bare Zn might be due to the severe H_2_ evolution and uncontrolled Zn dendrites growth. As revealed by the electrochemical impedance spectroscopy (EIS) analyses on the symmetric cells, Zn@Sb electrode exhibits a much lower charge‐transfer resistance (*R*_ct_) of 40.1 Ω, while that for the bare Zn reaches 704.8 Ω (Figure 4e), confirming the fast Zn^2+^ transfer kinetics of Zn@Sb anode. The rate performance of symmetric cells with the bare Zn and Zn@Sb anodes was evaluated at a spectrum of current densities (Figure 4f). Benefiting from the abundant zincophilic sites of Sb layer, Zn@Sb exhibits lower voltage hysteresis and more stable voltage plateau than bare Zn. Steady hysteresis of 17, 26, 28, 32, 45, and 64 mV are achieved for Zn@Sb at 0.2, 0.4, 1.0, 2.0, 4.0, and 10.0 mA cm^–2^, respectively, outperforming most of the Zn metal anodes reported in the literature (Figure 4g).^[^ ^1^, ^3^, ^11^ ^]^

The feasibility of Zn@Sb anodes in practical applications was further demonstrated by Zn||MnO_2_ full cells. The MnO_2_ cathode was fabricated through a hydrothermal method reported previously (Figure S6, Supporting Information).^[^ ^12^ ^]^ In the CV curves (Figure 4h), two pairs of characteristic redox peaks are detected for both Zn@Sb||MnO_2_ and bare Zn||MnO_2_ cells, in agreement with those reported in the literature.^[^ ^13^ ^]^ The Zn@Sb||MnO_2_ cells show higher current intensity and smaller voltage polarization than the Zn||MnO_2_ cells, indicating the enhanced electrochemical reactivity and reaction kinetics endowed by Sb protective layer. At 0.5 C (Figure S7, Supporting Information), the Zn@Sb||MnO_2_ cell delivers a high capacity of 276 mAh g^–1^, higher than that of the Zn||MnO_2_ cell (155 mAh g^–1^). In addition, the Zn@Sb||MnO_2_ cell shows lower *R*_ct_ (Figure S8, Supporting Information), which is conducive to the Zn^2+^ ions transfer at the electrolyte/anode interface. The long‐term cycling performance and corresponding CE of both cells are shown in Figure 4i. For Zn@Sb||MnO_2_ full cell, a capacity of 160 mAh g^–1^ is retained after 500 cycles at a high rate of 2 C, giving a retention rate of 75.5%. In contrast, the Zn||MnO_2_ cell exhibits fast capacity decay. A specific capacity of only 27 mAh g^–1^ maintains after 500 cycles. The enhanced cycling stability of the Zn@Sb||MnO_2_ cells is attributed to the suppression of the dendrite growth and hydrogen evolution reaction by the multifunctional Sb‐metal layer.

**Figure 5:**
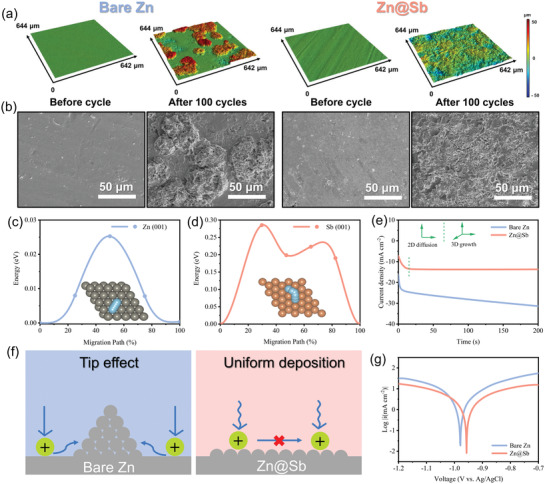
a) CLSM 3D height images and b) SEM images of bare Zn and Zn@Sb before cycling and after 100 cycles at 1.0 mA cm^–2^ with the capacity of 1.0 mAh cm^–2^. The calculated migration energy barriers of Zn^2+^ ions along c) Zn (001) and d) Sb (001) surface and corresponding migration pathways (insets). e) Chronoamperograms (CAs) of bare Zn and Zn@Sb at a −150 mV overpotential. f) Schematics of the Zn^2+^ diffusion and reduction processes on bare Zn and Zn@Sb electrodes. g) Corrosion curves of the bare Zn and Zn@Sb anodes.

The surface morphology evolution of the bare Zn and Zn@Sb anodes in the symmetric cells before and after cycling was revealed by CLSM and SEM observation (**Figure** **5**a,b). Before cycling, both the bare Zn and Zn@Sb anodes exhibit smooth surface with negligible altitude fluctuate. After 100 cycles, island‐like Zn dendrites with the altitude intercept drastically increased from 5.2  to 102 µm are observed on bare Zn surface (Figure 5a), attributing to the uncontrolled growth of Zn dendrites. However, the surface of Zn@Sb electrode is relatively smooth with a low altitude intercept of only ≈57 µm after 100 cycles, demonstrating the uniform plating process on the surface of Zn@Sb. The morphology evolution tendency was confirmed by SEM observation. The bare Zn anode exhibits a coarse surface with irregular and micrometer‐sized Zn dendrites after 100 cycles (Figure 5b and Figure S9, Supporting Information), while the Zn@Sb anode shows relatively homogeneous Zn deposition. The uniform Zn deposition induced by the Sb layer ensures the long‐term, dendrite‐free, and highly reversible cycling process.

Essentially, the dendrite growth is closely related to the diffusion behavior of Zn^2+^ ions on the electrode surface. The Zn diffusion pathways on Zn (001) and Sb (001) surface and the corresponding energy profiles were predicted by the DFT calculation (Figure 5c,d). On the Zn (001) surface, the diffusion barrier of Zn^2+^ ions is 0.025 eV, while that on the Sb (001) surface is as high as 0.286 eV, indicating that the migration of Zn^2+^ ions on Sb (001) surface is hindered and possibly suppressing the Zn aggregation.^[^ ^14^ ^]^ As a result, the Zn atoms on the Sb surface tend to lateral growth, while those on bare Zn surface prefer to form aggregation. The chronoamperometry (CA) analyses were further conducted to illustrate the Zn nucleation and growth mechanism (Figure 5e). The current variation in the CA curves is closely related to the mode of Zn deposition and the change in surface morphology. For the bare Zn anode, the current density continuously elevates within the duration of 200 s at an overpotential of −150 mV, indicating a rampant 2D diffusion process and accumulated Zn deposition.^[^ ^1a^ ^]^ Due to the low surface diffusion barrier (0.025 eV), the adsorbed Zn^2+^ ions can diffuse laterally along the surface and aggregate at the most energetically favorable sites, thus inducing the growth of Zn dendrite (Figure 5f). For the Zn@Sb anode, after the initial Zn nucleation process (within 15 s), the current density reaches a steady state, indicating a stable 3D diffusion procedure on the surface of Zn@Sb. The 2D surface diffusion is constrained by the high Zn^2+^ migration barrier. Thus, Zn^2+^ ions are absorbed on the zincophilic sites of the electrode surface and reduced to Zn^0^, consequently inducing uniform Zn deposition. The corrosion properties of the Sb layer were revealed by the linear polarization experiments (Figure 5g). The corrosion current density of Zn@Sb is ≈3.90 mA cm^–2^, much lower than that of bare Zn (7.61 mA cm^–2^), demonstrating the improved corrosion resistance properties of Zn@Sb with the coated Sb protective layer.

**Figure 6:**
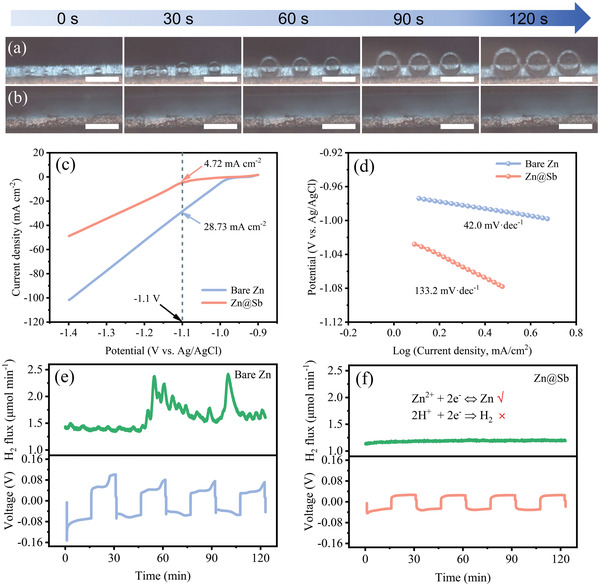
In situ optical microscopy observation of a) bare Zn and b) Zn@Sb in a transparent symmetric cell with an applied current density of at 0.5 mA cm^–2^. Scale bars: 200 µm. c) Hydrogen evolution polarization curves of bare Zn and Zn@Sb in 2.0 m ZnSO_4_ and d) corresponding Tafel slope curves. In situ DEMS analysis of hydrogen evolution flux for symmetric cells with e) bare Zn and f) Zn@Sb.

The direct evidences for restrained H_2_ evolution on the Zn@Sb electrode were obtained from in situ optical microscopy observation in transparent symmetric cells. Hydrogen bubbles were observed on the surface of bare Zn even the cells were at the open‐circuit state. With a current density of 0.5 mA cm^–2^, these initial bubbles gradually grow, merge, and collapse within 120 s in ZnSO_4_ aqueous electrolyte (**Figure** **6**a). Impressively, no hydrogen bubbles were detected on Zn@Sb under the same conditions (Figure 6b), demonstrating that H_2_ evolution reaction can be well alleviated by the introduction of Sb protective layer. Furthermore, the H_2_ evolution polarization experiments of both electrodes were performed in 2.0 m ZnSO_4_ electrolyte (Figure 6c). The hydrogen evolution current density of Zn@Sb is much lower than that of bare Zn in a wide voltage range (−0.9 to −1.4 V vs Ag/AgCl). Particularly, the H_2_ evolution current density of bare Zn is 28.73 mA cm^−2^ at −1.1 V, while that of Zn@Sb decreases to 4.72 mA cm^−2^, further verifying the H_2_ evolution suppression of Zn@Sb electrode. Correspondingly, Zn@Sb shows a Tafel slope of 133.2 mV dec^–1^, higher than that of bare Zn plate (42.0 mV dec^–1^) (Figure 6d). The high Tafel slope further indicates the sluggish hydrogen generation rate endowed by Sb layer. In order to accurately quantify the amount of H_2_ generated, differential electrochemical mass spectrometry (DEMS) was employed to in situ detect the hydrogen flux of symmetric cells during plating/stripping process. For bare Zn anode, the H_2_ flux appears drastic fluctuation and reaches a maximum H_2_ evolution rate of 2.42 µmol min^–1^, suggesting that the reduction process of Zn competes with H_2_ evolution reaction (Figure 6e). With the protection of Sb‐metal layer, the amount of hydrogen release is negligible during the repeated plating/stripping process (Figure 6f).

## 3 Conclusions

To address the issues of the uncontrolled dendrite growth on Zn metal anodes and the hydrogen evolution reaction of rechargeable aqueous Zn‐ion batteries, the formation of an efficient zincophilic Sb protective layer on the surface of Zn anodes was designed based on the DFT calculation and achieved by in situ replacement reaction. The low binding energy between Sb and Zn enhances the interfacial interaction between Zn^2+^ ions and Zn@Sb. With rich zincophilic sites, improved electrolyte wettability and uniformized electric field brought by the Sb layer, homogeneous distribution of Zn^2+^ ions and charge and repeatable plating/stripping were achieved, leading to the uniform nucleation/deposition of Zn on the anode. Furthermore, as demonstrated by in situ quantification analyses, hydrogen evolution reaction was effectively suppressed by the Sb protective layer. As a result, the symmetric cells assembled with Zn@Sb anodes exhibited excellent cycling stability for over 1000 h with a low voltage hysteresis of 34 mV at 3 mA cm^–2^ for 1.0 mAh cm^–2^, achieving both dendrite‐free morphology and hydrogen‐free behavior. When coupled with MnO_2_, the Zn@Sb||MnO_2_ full cells delivered impressive cycling stability with a high capacity retention of 75.5% after 500 cycles at 2 C. This work provides a simple and efficient strategy for the development of high‐performance Zn anodes with both dendrite‐ and hydrogen‐free behavior through the formation a thin metal layer on the Zn metal anodes by facile replacement reactions.

## 4 Experimental Section

### Preparation of Zn@Sb

Zn foils (purity 99.99%, ≈0.1mm) were washed with hydrochloric acid and subsequently with deionized water and ethanol for several times to remove surface impurities before use. The clean Zn foils were immersed into 100 mL of 0.15 m SbCl_3_ ethanol solution under stirring at room temperature for 5 min. The treated Zn foils were washed with ethanol for several times to remove the residual reagents and dried at 90 °C for 12 h under vacuum.

### Characterizations

XRD patterns were collected by a Rigaku Mini Flex 600 diffractometer using Cu K*α*‐radiation (*λ* = 1.5418). The morphology of the samples was observed on a field emission scanning electron microscope (SEM, Nova NanoSEM 450) equipped with an energy dispersive X‐ray spectrometer. The CLSM images were collected on Olympus OLS5000 microscope. The in situ optical visualization observation of Zn plating/stripping behavior was performed on an optical microscope (LEICA DM 4000). The wettability of electrodes was performed by a contact angle measuring device (DSA30, KRUSS). In situ DEMS tests were conducted on a commercial mass spectrometer (QAS 100) with a sealed Swagelok‐type cell containing two pieces of Zn anodes, a glass fiber separator, and electrolyte (2.0 m ZnSO_4_, 200 µL). The flow rate for ultrapure Ar is 0.5 mL min^−1^.

### Electrochemical Measurements

CR2032‐type Zn–Zn symmetric cells were assembled with bare Zn and Zn@Sb (diameter: 12 mm, thickness: 0.1 mm), glass fiber separator, and 2.0 m ZnSO_4_ electrolyte. The cathodes were fabricated with a slurry containing MnO_2_, acetylene black and polyvinylidene fluoride (PVDF) at a weight ratio of 8:1:1. The cathodes and anodes were separated by glass fiber separators (*Φ* = 19 mm). 2.0 m ZnSO_4_ and 0.1 m MnSO_4_ aqueous solution was used as electrolyte for all coin cells, which were assembled in the air atmosphere. The galvanostatic charge/discharge tests were performed on a Neware battery testing system. CV, EIS, and corrosion tests were carried out on an electrochemical workstation (CHI760E, China).

### Computational Method

DFT calculations were carried out using projector‐augmented wave (PAW) method as implemented in Vienna ab initio simulation package (VASP).^[^ ^15^ ^]^ A generalized gradient approximation (GGA) of Perdew–Burke–Ernzerhof (PBE) functional was employed to describe the exchange‐correlation interaction.^[^ ^16^ ^]^ An energy cutoff of 500 eV and Gamma centered 3 × 3 × 1 *k*‐points mesh were applied to all calculations. To simulate the interaction between the deposited Zn atom and electrode, the Zn (0001) and Sb (0001) surfaces were cleaved from the corresponding crystal structure as the most stable surface, and a vacuum layer of 20 Å was adopted. The structures were relaxed until the forces and total energy on all atoms were converged to less than 0.05 eV Å ^−1^ and 1 × 10 ^−5^ eV, respectively. To evaluate the interaction between Zn atom and the electrode, the binding energy (*E*_b_) was calculated as follows

(1) $$E_{b}=E_{\mathrm{Total}}\;-E_{\mathrm{Zn}}-E_{\mathrm{Surface}}$$

where *E*_Surface_ and *E*_Total_ are the total energy of compound before and after Zn adsorption, respectively. *E*_Zn_ is the energy of a single Zn atom. The lower the binding energy, the stronger the interaction between Zn and electrode. Activation barriers for deposited Zn hopping between adjacent interstitial sites on the surface were calculated using the climbing‐image nudged elastic band (CI‐NEB) method.^[^ ^17^ ^]^

Hydrogen adsorption ∆*G*_H_ was calculated as

(2) $$\Delta G_{H}=\Delta E_{\mathrm{DFT}}+\Delta E_{\mathrm{ZPE}}-T\Delta S$$

where ∆*E*_DFT_, ∆*E*_ZPE_, and *T*∆*S* denote the DFT calculated adsorption energy, change of zero point energy and change of entropic contribution, respectively. TS term for H adsorbate is considered negligible, and *T*∆*S* ≈ −0.5*S*_H2_ = −0.24 eV.

### Electric Field Simulation

A simplified 2D parallel plate capacitor model was established to simulate the electric field distribution at the anode/electrolyte interface based on COMSOL Multiphysics software. In this model, the length of two electrodes is 5.0 µm and the height is 0.3 µm with a distance of 3.3 µm between them. The protuberances on bare Zn and Sb nanoparticles on Zn@Sb were represented by semi‐ellipse and semicircles, respectively. The size of protuberances and Sb nanoparticles were based on the SEM results. The voltage hysteresis from symmetric cells was set as cathodic potential and the anodic potential was a constant of 0.

## Conflict of Interest

The authors declare no conflict of interest.

## Supporting information

Supporting Information

## Data Availability

The data that support the findings of this study are available from the corresponding author upon reasonable request.
