# Supplementary material for: Toward Hydrogen‐Free and Dendrite‐Free Aqueous Zinc Batteries: Formation of Zincophilic Protective Layer on Zn Anodes
Source: Adv Sci (Weinh). 2022 Jan 6;9(6):2104866. doi: 10.1002/advs.202104866 (PMC8867158; doi:10.1002/advs.202104866)
Supplement: Supplementary file 1 — Supporting Information [file ADVS-9-2104866-s001.pdf]

ADVANCED SCIENCE
Open Access

Supporting Information

for *Adv. Sci.,* DOI: 10.1002/advs.202104866

Towards Hydrogen-Free and Dendrite-Free Aqueous Zinc Batteries: Formation of Zincophilic Protective Layer on Zn Anodes

Lin Hong, Liang-Yu Wang, Yuling Wang, Xiuming Wu, Wei Huang, Yongfeng Zhou, Kai-Xue Wang,* Jie-Sheng Chen*

Supporting Information

Towards Hydrogen-Free and Dendrite-Free Aqueous Zinc Batteries: Formation of Zincophilic Protective Layer on Zn Anodes

Lin Hong, Liang-Yu Wang, Yuling Wang, Xiuming Wu, Wei Huang,* Yongfeng Zhou, Kai-Xue Wang,* Jie-Sheng Chen

School of Chemistry and Chemical Engineering, State Key Laboratory of Metal Matrix Composites, Shanghai Jiao Tong University, 800 Dongchuan Road, Shanghai 200240, P. R. China.

E-mail: hw66@sjtu.edu.cn (WH); k.wang@sjtu.edu.cn (KXW)

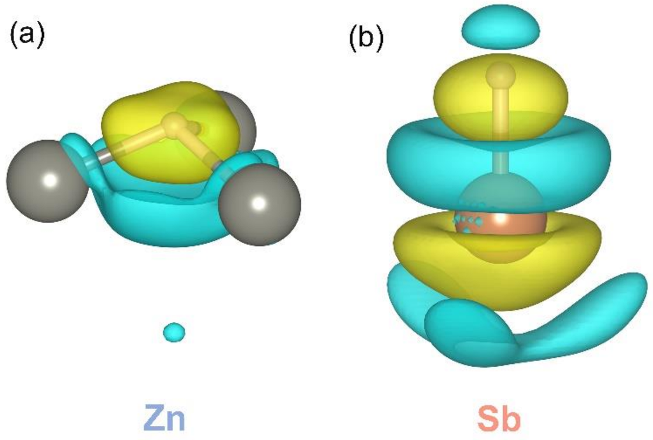

Figure S1 Adsorption configuration of H adsorbed on (a) Zn and (b) Sb.

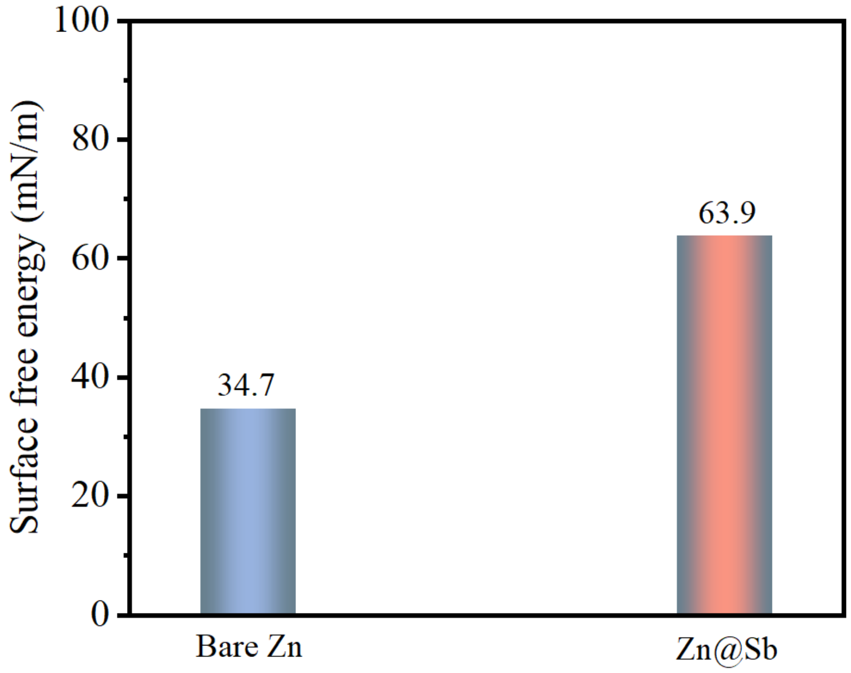

Figure S2 Surface free energy on bare Zn and Zn@Sb.

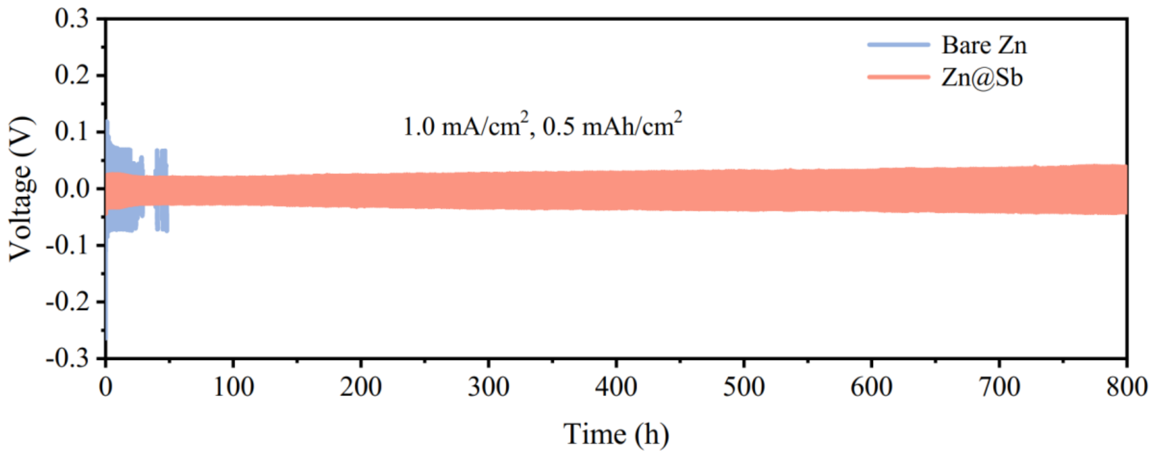

Figure S3 Cycling performance of the symmetric cells with the Zn@Sb and bare Zn anodes for 0.5 mA h/cm^2 at 1.0 mA/cm^2.

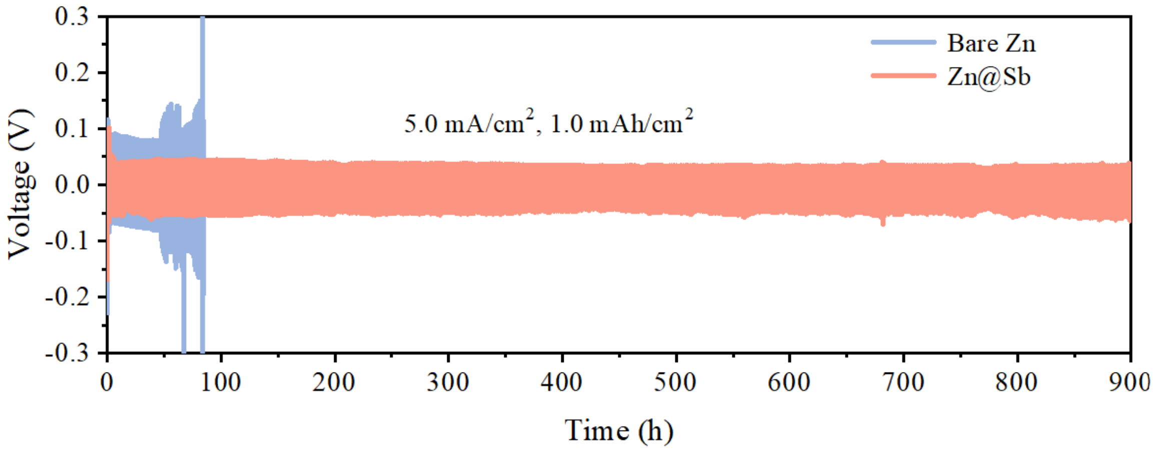

Figure S4 Cycling performance of the symmetric cells at (a) 5.0 mA/cm^2 for 1.0 mA h/cm^2.

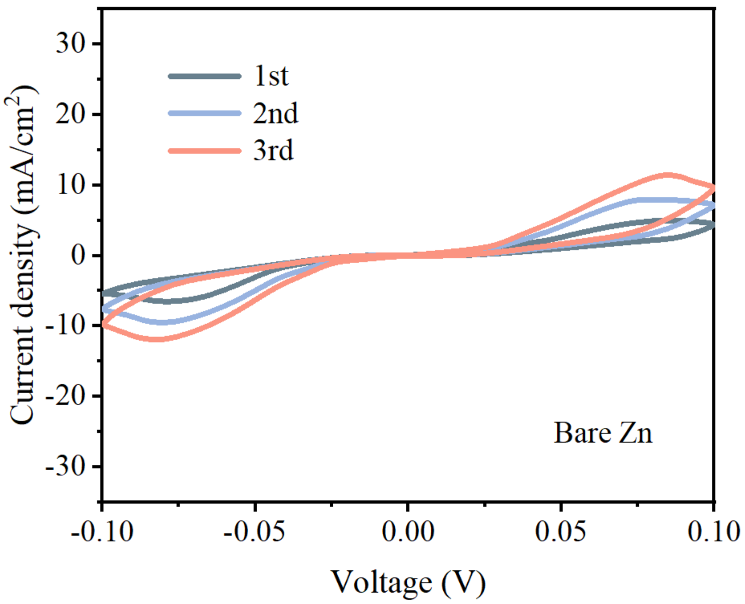

Figure S5 CV curves of symmetric cells with bare Zn measured at 0.1 mV s^{-1}.

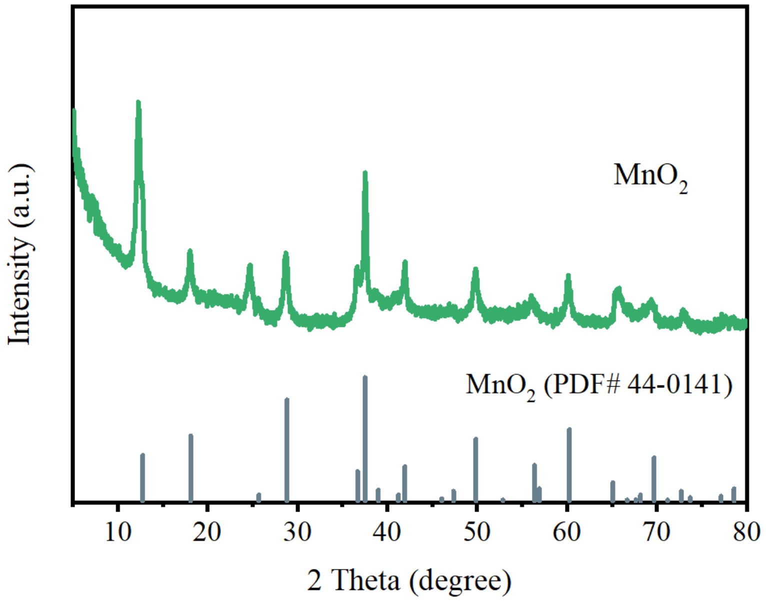

Figure S6 XRD pattern of MnO_2.

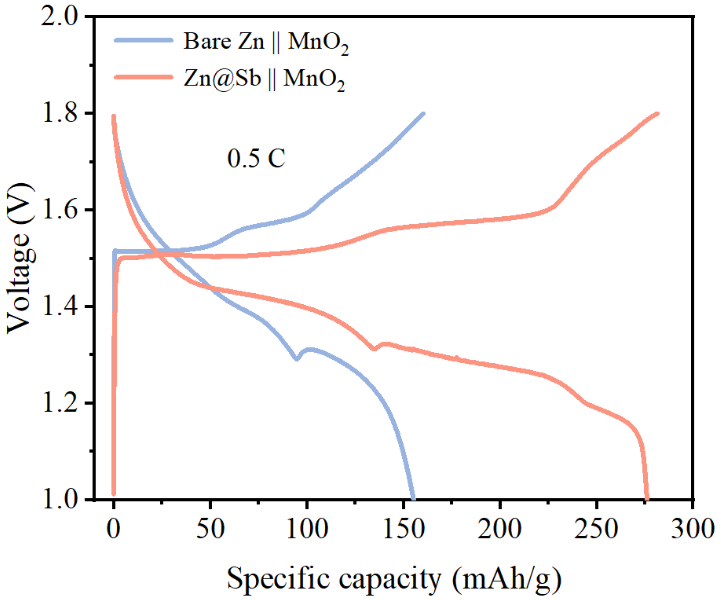

Figure S7 Charge/discharge curves of the Zn||MnO_2 and Zn@Sb||MnO_2 full cells at 0.5 C.

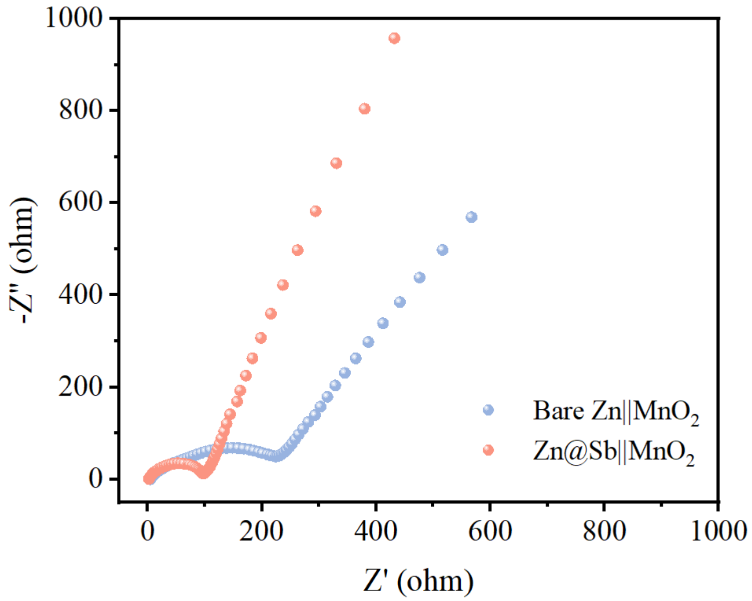

Figure S8 Nyquist plots of the Zn||MnO_2 and Zn@Sb||MnO_2 full cells before cycling.

Table S1 Summary of the performance of the symmetric cells and full cells.

Anode	Symmetric cell performance			Full cell performance				Ref.
	Current density (mA/cm^2)	Capacity (mAh/cm^2)	Life (h)	Cathode	Current density	Capacity (mAh/g)	Cycle number	**Ref.**
**Zn@Sb**	**5**	**1**	**900**	**MnO_2**	**2 C**	**160**	**500**	**This work**
ZF@F-TiO_2	1	1	460	MnO_2	1 A/g	80	300	[1]
Zn/Sn_{200}	1	1	500	-	-	-	-	[2]
$CaCO_3$ coated Zn	2	0.1	80	MnO_2	1 A/g	175	1000	[3]
Zn/ZCO-30	1	1	1400	MnO_2	0.5 A/g	165	200	[4]
Zn@CFs	1	1	160	MnO_2	1 C	207	140	[5]
502-coated Zn	2	1	400	$V_2O_5 \cdot 1.6H_2O$	1 A/g	95	550	[6]
NTP@Zn	1	1	250	MnO_2	5 C	128	600	[7]
Zn@N-VG@CC	1	1	65	MnO_2	2 A/g	250	300	[8]
MZn-60	0.2	0.2	800	MnO_2	1 A/g	205	500	[9]
CM@CuO@CC	1	1	340	MnO2	5 A/g	140	900	[10]

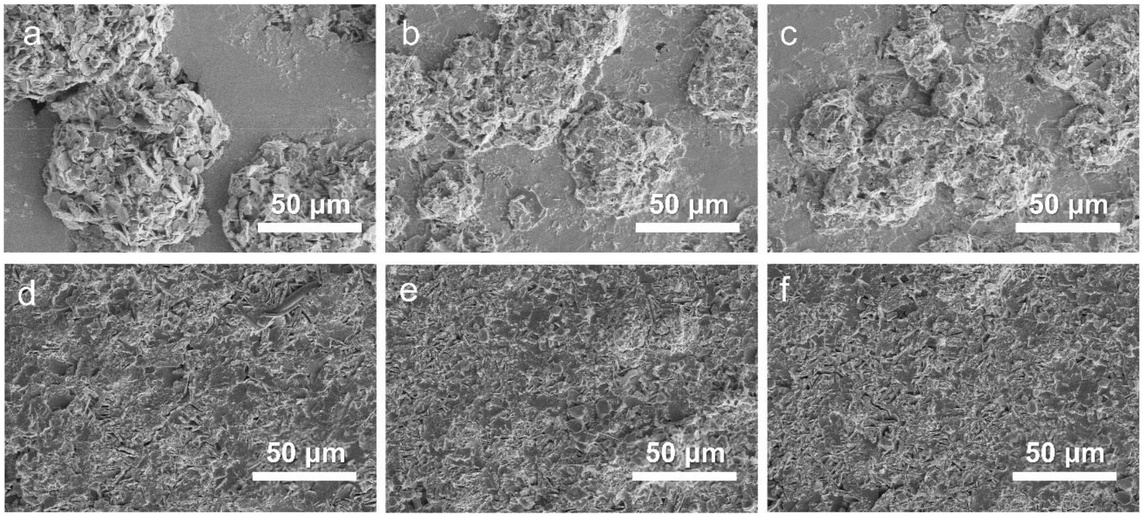

Figure S9 SEM images of (a-c) bare Zn and (d-f) Zn@Sb after 100 cycles at (a and d) 1.0 mA/cm^2 for 0.5 mA h/cm^2, (b and e) 3.0 mA/cm^2 for 1.0 mA h/cm^2 and (c and f) 5.0 mA/cm^2 for 1.0 mA h/cm^2.

References

[1] Q. Zhang, J. Luan, X. Huang, Q. Wang, D. Sun, Y. Tang, X. Ji and H. Wang, *Nat. Commun.*, **2020**, *11*, 3961.

[2] S. Li, J. Fu, G. Miao, S. Wang, W. Zhao, Z. Wu, Y. Zhang, X. Yang, *Adv. Mater.*, **2021**, *33*, 2008424.

[3] L. Kang, M. Cui, F. Jiang, Y. Gao, H. Luo, J. Liu, W. Liang and C. Zhi, *Adv. Energy Mater.*, **2018**, *8*, 1801090.

[4] X. Xu, Y. Chen, D. Zheng, P. Ruan, Y. Cai, X. Dai, X. Niu, C. Pei, W. Shi, W. Liu, F. Wu, Z. Pan, H. Li, X. Cao, *Small*, **2021**, *17*, 2101901.

[5] W. Dong, J. Shi, T. Wang, Y. Yin, C. Wang and Y. Guo, *RSC Adv.*, **2018**, *8*, 19157-19163.

[6] Z. Cao, X. Zhu, D. Xu, P. Dong, M. Chee, X. Li, K. Zhu, M. Ye, J. Shen, *Energy Storage Mater.*, **2021**, *36*, 132-138.

[7] M. Liu, J. Cai, H. Ao, Z. Hou, Y. Zhu and Y. Qian, *Adv. Funct. Mater.*, **2020**, *30*, 2004885.

[8] Q. Cao, H. Gao, Y. Gao, J. Yang, C. Li, J. Pu, J. Du, J. Yang, D. Cai, Z. Pan, C. Guan, W. Huang, *Adv. Funct. Mater.*, **2021**, 2103922.

[9] N. Zhang, S. Huang, Z. Yuan, J. Zhu, Z. Zhao and Z. Niu, *Angew. Chem., Int. Ed.*, **2021**, *60*, 2861-2865.

[10] Q. Zhang, J. Luan, X. Huang, L. Zhu, Y. Tang, X. Ji and H. Wang, *Small*, **2020**, *16*, 2000929.
